# Reducing bed rest time from five to three hours does not increase complications after cardiac catheterization: the THREE CATH Trial**^1^**

**DOI:** 10.1590/1518-8345.0725.2796

**Published:** 2016-07-25

**Authors:** Roselene Matte, Thamires de Souza Hilário, Rejane Reich, Graziella Badin Aliti, Eneida Rejane Rabelo-Silva

**Affiliations:** 2 RN, MSc, Hospital de Clínicas de Porto Alegre, Porto Alegre, RS, Brazil.; 3 RN.; 4 PhD, Adjunct Professor, Escola de Enfermagem, Universidade Federal do Rio Grande do Sul, Porto Alegre, RS, Brazil.; 5 PhD, Associate Professor, Escola de Enfermagem, Universidade Federal do Rio Grande do Sul, Porto Alegre, RS, Brazil.

**Keywords:** Cardiac Catheterization, Bed Rest, Early Ambulation, Hematoma, Nursing Care

## Abstract

**Objective:**

to compare the incidence of vascular complications in patients undergoing transfemoral cardiac catheterization with a 6F introducer sheath followed by 3-hour versus 5-hour rest.

**Methods:**

randomized clinical trial. Subjects in the intervention group (IG) ambulated 3 hours after sheath removal, versus 5 hours in the control group (CG). All patients remained in the catheterization laboratory for 5 hours and were assessed hourly, and were contacted 24, 48, and 72 h after hospital discharge.

**Results:**

the sample comprised 367 patients in the IG and 363 in the GC. During cath lab stay, hematoma was the most common complication in both groups, occurring in 12 (3%) IG and 13 (4%) CG subjects (P=0.87). Bleeding occurred in 4 (1%) IG and 6 (2%) CG subjects (P=0.51), and vasovagal reaction in 5 (1.4%) IG and 4 (1.1%) CG subjects (P=0.75). At 24-h, 48-h, and 72-h bruising was the most commonly reported complication in both groups. None of the comparisons revealed any significant between-group differences.

**Conclusion:**

the results of this trial show that reducing bed rest time to 3 hours after elective cardiac catheterization is safe and does not increase complications as compared with a 5-hour rest. ClinicalTrials.gov Identifier: NCT-01740856

## Introduction

The rate of complications after femoral artery puncture for coronary angiography or diagnostic cardiac catheterization ranges from 1.5% to 3.7%, with vascular complications having the highest incidence^1^. Particularly in the first 6 to 12 hours after a transfemoral procedure, care of the insertion site is a determining factor in the occurrence or mitigation of these complications and a reminder of the constant vigilance that these patients require^2^. 

Despite rapid advancement in techniques, catheters, contrast agents, and implantable devices, post-catheterization nursing care has not evolved at a similar pace, and remains classically based on bed rest, which may last 2 hours^3^ to 24 hours^4^. A recent meta-analysis of 20 studies and 4,019 patients found that a bed rest duration of 2-3 hours after transfemoral catheterization is safe, has no effect on the incidence of vascular complications, and may reduce back pain and discomfort^5^.

Although the literature suggests that early mobilization is safe after diagnostic catheterization, studies are unclear as to the setting of this intervention. Furthermore, the only study on shorter bed rest conducted in Brazil was carried out at a private clinic^6^; therefore, its results cannot be extrapolated to a large, high-complexity teaching hospital, which was the proposed setting for the present study. In addition, at the facility where the present study was carried out, transfemoral diagnostic catheterization is performed by multiple operators, ranging from experienced physicians to residents in training, and is followed by a standard 5-hour rest period. This study is relevant insofar as it reports the results of an intervention that could be incorporated immediately into the clinical practice of a variety of health facilities with similar profiles. 

Within this context, the present randomized controlled trial (RCT) was designed to test the hypothesis that reducing the duration of bed rest to 3 hours in the intervention group (IG) from 5 hours in the control group (CG) would not increase the rate of arterial puncture-related complications after elective transfemoral diagnostic cardiac catheterization with a 6F introducer sheath.

## Methods

This is a report of the THREE CATH (*Reducing rest time to THREE hours after cardiac CATheterization does not increase complications related to the procedure*) RCT, registered at ClinicalTrials.gov with accession number NCT-01740856, with blinded outcome assessment. This trial was conducted at the catheterization laboratory (cath lab) of a public university hospital located in the greater Porto Alegre area, state of Rio Grande do Sul, Brazil, from January 2011 to September 2013. 

### Participants

The study sample comprised adult outpatients who underwent elective transfemoral diagnostic cardiac catheterization with a 6F introducer sheath. The exclusion criteria were any restrictions to ambulation, use of coumarin anticoagulants, body mass index (BMI) > 35kg/m^2^, hypertension with a systolic blood pressure (SBP) > 180mmHg or diastolic blood pressure (DBP) > 110mmHg at the end of the procedure, and a history of uncontrolled bleeding. 

The study protocol was approved by the Research Ethics Committee of the facility where the trial was carried out. All patients were informed of the objectives of the study and were only included after having read and signed an informed consent form.

### Study protocol and group allocation

Patients who met the inclusion criteria and were considered eligible were invited to take part in the study. At the end of the procedure, patients were taken to the observation unit (OU). Removal of the introducer sheath with hemostasis valve and manual (digital) compression of the insertion site for 15 minutes were performed by the nursing staff in both groups. Patients were instructed to refrain from moving the affected leg. After the second hour of bed rest, the nursing team contacted the unit secretary to be informed of patient randomization. All interventions were performed by the nursing staff, which had been duly trained to carry out all actions in a consistent manner in accordance with the study protocol.

Data on pre- and post-procedure clinical condition and clinical parameters of interest (sex, age, BMI, diabetes, hypertension, peripheral vascular disease, current antiplatelet therapy) from patients in both groups (IG and CG) were recorded.

### Intervention group

Patients randomly allocated to the intervention group (IG) remained in bed, in the supine position, for 2 hours after completion of manual (digital) compression. After this period, the nursing staff initiated the intervention by elevating the head of bed at 45 degrees for 60 minutes and making the patients ambulate around the cath lab for approximately 10 minutes. The staff than instructed patients to remain seated out of bed in the cath lab until the end of the 5-hour rest period, at which time patients were discharged and walked out of the hospital.

### Control group

Participants randomly allocated to the CG remained in bed, in the supine position, for 4 hours after completion of the manual compression period. After this period, patients remained with the head of bed elevated at 45 degrees for 60 minutes and were made to ambulate around the cath lab for 10 minutes. Patients were then discharged and walked out of the hospital.

Both groups received post-procedural care guidance and were monitored hourly by the nursing staff. Patients were notified that they would be contacted by telephone 24, 48, and 72 hours after hospital discharge, and were given an instruction sheet containing descriptions and illustrative images of bleeding, hematoma, bruising, and pseudoaneurysm, as well as a ruler, with which they could measure any visible complications at the puncture site. 

### Primary outcome

The primary outcome was occurrence of complications (hematoma, bleeding, and pseudoaneurysm), defined as follows:

1) Hematomas at the arterial puncture site, classified in accordance with the American College of Cardiology definition (large, > 10 cm; small, < 10 cm)^7^;
2) Major bleeding, as defined for the *Evaluating the Performance of the Can Rapid Risk Stratification of Unstable Angina Patients Suppress Adverse Outcomes With Early Implementation of the ACC/AHA Guidelines (CRUSADE) bleeding score* trial: documented retroperitoneal bleeding (not requiring surgical correction) or any red blood cell transfusion with witnessed bleed^8^. Patients who developed hemodynamic instability, defined as uncontrolled hypertension or hypotension, tachycardia or bradycardia, or desaturation from baseline, were also considered to have major bleeding. If no hemodynamic instability occurred, bleeding was considered minor^8^;
3) Any of the following vascular complications requiring surgical correction: retroperitoneal bleeding, pseudoaneurysm, or development of arteriovenous fistula^9^.

### Secondary outcomes

The secondary outcomes of interest were vasovagal response to sheath withdrawal, bruising, association/comparison between current medications and comorbidities, and association/comparison between sex and events during OU stay and at 24-, 48-, and 72-hour follow-up.

### Sample size calculation

The sample size calculation was based on the assumption that the proportion of events would not be higher in IG than in CG participants. Considering a negligible difference between the intervention and control group, a 2% rate of events, and a 20% attrition rate, the minimum sample size was estimated at 714 patients for an alpha level of 0.05 and 80% statistical power^9^. Overall, 48 participants (6.6%) were lost to follow-up, for a final sample size of 730.

### Randomization

The website www.randomization.com was used to generate a simple random numbers list, which was managed by a person not involved in the trial (cath lab secretary) responsible for patient allocation into IG and CG. Participants were always randomized at hour 2 of bed rest at the OU after the procedure. At that time, the cath lab secretary was instructed by the nursing team to check the randomization list and state the group to which the patient should be allocated.

### Blinding

The interventional radiology team and cath lab nursing staff were blinded to group allocation until the second hour of bed rest. Outcome monitoring during OU stay was carried out by the nursing team, whereas outcome assessment by telephone was performed by a provider blinded to group allocation.

### Statistical analysis

Variables were entered into a Microsoft Excel spreadsheet and analyzed in PASW Statistics 18*.* Continuous variables were expressed as means and standard deviations if normally distributed, and categorical variables were expressed as absolute number and relative frequencies. Student's *t-*test was used for between-group comparisons of normally distributed quantitative variables. Pearson's chi-square test was used with the same purpose for categorical variables, as well as to test for potential associations of current medications and comorbidities with occurrence of the events of interest. Relative risks with respective 95% confidence intervals were calculated to assess the effect size of the intervention. Two-tailed P-values < 0.05 were considered significant.

## Results

**Figure 1:**
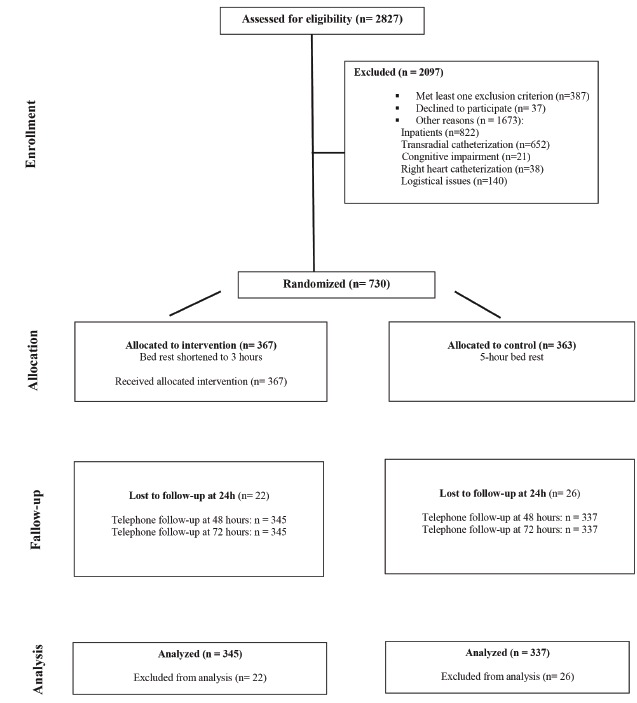
Consolidated Standards of Reporting Trials (CONSORT) flow diagram

From January 2011 to September 2013, 2,827 patients underwent elective diagnostic catheterization with a 6F introducer sheath at the study facility. Of these, 387 met at least one of the exclusion criteria and were thus left out of the sample. High blood pressure at the time of randomization (SBP ≥ 180mmHg or DBP ≥ 110mmHg at the end of the procedure) was the leading reason for exclusion, followed by motor issues that hindered ambulation and, at lower rates, obesity and use of coumarin anticoagulants and heparin. Thirty-seven patients dropped out of the study, and 1,673 were excluded for other reasons, including hospitalization at the time of diagnostic catheterization (n=669, 40%), transradial catheterization (n=602, 36%) and, at lower rates, cognitive impairment, right heart catheterization, and logistical issues. Overall, 730 participants were randomized: 367 into the IG and 363 into the CG (Figure 1).

## Sample profile

Table 1 illustrates the profile of the intervention and control groups at baseline. Mean age was similar in both groups, and both were composed predominantly of female participants. Diabetes mellitus (DM) and hypertension (HTN) were the most prevalent comorbidities. Overall, the groups were homogeneous for all variables. 

**Table 1:** Demographic and clinical profile of patients undergoing diagnostic cardiac catheterization with a 6F introducer sheath. Porto Alegre, RS, Brazil, 2013

Variable	Overall (n=730)	Intervention group (n=367)	Control group (n=363)	P
Age, years	62 ± 11	61.5 ± 11	63 ± 10	0.14^*^
Females, n (%)	407 (56)	211 (57.5)	196 (54)	0.34 ^†^
Weight, kg	76 ± 14	75 ± 14	76 ± 13	0.76^*^
Height, cm	163 ± 10	163 ± 10	164 ± 9	0.32^*^
Body mass index, kg/m^2^	28 ± 4	28 ± 4	28 ± 4	0.90^*^
Diabetes, n (%)	245 (34)	131 (36)	114 (31)	0.22 ^†^
Hypertension, n (%)	616 (84)	310 (84.5)	316 (84)	0.94 ^†^
Peripheral vascular disease, n (%)	23 (3)	14 (4)	9 (2.5)	0.30 ^†^
Systolic blood pressure, mmHg	145 ± 25	145 ± 25	145 ± 25	0.74^*^
Diastolic blood pressure, mmHg	82 ± 13	81 ± 13	82 ± 13	0.49^*^
Current medications, n (%)				
Aspirin	515 (70)	252 (69)	263 (72)	0,74^†^
Clopidogrel	153 (21)	74 (20)	79 (22)	059^†^

±: standard deviation; *: Student's t-test; n (%): categorical variables; †: chi-square test.

### Complications detected during observation unit stay

Table 2 lists the post-procedural complications observed throughout the OU stay period. Hematoma was the most common complication in both groups, with no significant difference. Relative risks and confidence intervals for hematoma and bleeding were RR = 0.91 (95%CI 0.42-1.97) and RR = 0.66 (95%CI 0.19-2.32) respectively. The relative risk of vasovagal response was RR = 1.24 (95%CI 0.34-4.57). No comparison was statistically significant. None of the participants developed pseudoaneurysm or any other vascular complications during the observation period.

**Table 2:** Complications after diagnostic cardiac catheterization detected during observation. Porto Alegre, RS, Brazil, 2013

Complication	Overall (n = 730)	Intervention group (n = 367)	Control group (n = 363)	*P	RR (95%CI)
Hematoma, n (%)	25 (3.4)	12 (3.3)	13 (3.6)	0.87	0.91 (0.42-1.97)
Bleeding, n (%)	10 (1.4)	4 (1.1)	6 (1.7)	0.51	0.66 (0.19-2.32)
Vasovagal response, n (%)	9 (1.2)	5 (1.4)	4 (1.1)	0.75	1.24 (0.34-4.57)

*P: Pearson's chi-square test; RR: relative risk; 95%CI: confidence interval.

### Complications detected at 24-hour, 48-hour, and 72-hour follow-up

At 24-, 48-, and 72-hour telephone follow-up, the majority of participants in both groups were complication-free. Overall, 48 participants (7%) were lost to 24-hour follow-up (22 IG and 26 CG). Thus, 345 IG group and 337 CG participants remained for analysis and were contacted at 48 hours and 72 hours. 

Bruising was the most prevalent complication at all three time points in both groups, followed by pain at insertion site (Table 3). Hematoma was the third most prevalent complication during this period. Only one participant (0.3%), allocated to the CG, had developed a pseudoaneurysm at 48-hour follow-up and required in-hospital treatment. There were no significant between-group differences in prevalence of any of the complications assessed.

**Table 3:** Complications after diagnostic cardiac catheterization detected during home follow-up at 24, 48, and 72 hours. Porto Alegre, RS, Brazil, 2013

Complication	24 hours n (%)	48 hours n (%)	72 hours n (%)
None Intervention group	206 (59.4)	203 (58.8)	204 (59.1)
Control group	200 (59.3)	199 (59.1)	205 (60.8)
Bruising			
Intervention group	102 (29.6)	112 (32.5)	116 (33.6)
Control group	97 (28.8)	113 (33.5)	114 (33.8)
Pain			
Intervention group	29 (8.4)	23 (6.7)	19 (5.5)
Control group	33 (9.8)	21 (6.2)	14 (4.2)
Hematoma			
Intervention group	8 (2.3)	7 (2.0)	6 (1.7)
Control group	7 (2.1)	3 (0.9)	3 (0.9)
P^*^	0.841	0.619	0.612

*P: Pearson's chi-square test

### Associations between antiplatelet therapy or comorbidities and development of complications

Among patients who were on clopidogrel or aspirin, use of these agents was not associated with complications. Likewise, the presence of HTN, DM, or peripheral vascular disease was not associated with any of the events of interest.

### Associations between sex and development of complications during observation unit stay and at 24-hour, 48-hour, and 72-hour follow-up

During OU stay, event rates were similar in male and female participants, regardless of group (P=0.250). At 24-, 48-, and 72-hour telephone follow-up, the pooled event rate (bruising, pain, hematoma) was significantly higher in women (n=156, 41%) than in men (n=92, 30%) (P=0.004). Between-group comparison of the occurrence of events during OU stay showed no significant difference between IG and CG (P=0.691). This finding remained unchanged during follow-up in both groups (P=0.888). 

## Discussion

This RCT was the first study conducted in a public teaching hospital in Latin America to test the hypothesis that reducing bed rest from 5 to 3 hours after transfemoral diagnostic catheterization with a 6F introducer sheath would be safe and would not increase the rate of arterial puncture-related complications. 

As a result of reducing the duration of bed rest from 5 to 3 hours, involving multiple operators with different learning curves for arterial puncture and insertion site hemostasis, there was no increase in the rate of hematoma, bleeding, pseudoaneurysm, vasovagal response, or other complications during OU stay. Likewise, at 24-hour, 48-hour, and 72-hour telephone follow-up, the majority of participants were free of complications. Among patients who were on clopidogrel or aspirin, use of these medicines was not associated with occurrence of complications. The presence of comorbidities was also not associated with increased risk of complications at any time during follow-up. 

In both groups, the most common vascular complication during OU stay was puncture site hematoma (no significant between-group difference), followed by bleeding and vasovagal response. However, at 24-hour, 48-hour, and 72-hour telephone follow-up, it was the complication least reported by patients. The literature suggests that the incidence of arterial access-related hematoma ranges from 0.1 to 9%, with hematomas graded as large if > 10cm or small if < 10cm^10^. 

In a meta-analysis of 20 studies and 4,019 patients that sought to assess the effects of bed rest duration after transfemoral cardiac catheterization, the incidence of hematoma was approximately 7.6%^5^. It should be noted that the included studies employed different introducer sheath sizes, ranging from 4F to 9F. Some authors have reported that female patients are more prone to developing hematoma^6^ ^,^ ^11^ ^-^ ^12^. Characteristics such as body surface area, vessel size, increased sensitivity to anticoagulants and antiplatelet agents, or hormonal differences may explain this predilection^12^ ^-^ ^13^. In the present RCT, regardless of group allocation, women reported significantly more events than men at 24-hour, 48-hour, and 72-hour telephone follow-up, which corroborates the existing literature.

Puncture site bleeding was the second most common complication in both groups during OU stay; conversely, no cases were reported at 24-hour, 48-hour, and 72-hour telephone follow-up. All bleeding episodes occurred in participants still resting in bed at the OU and as soon as participants began to ambulate, after the time frame stipulated for each group. Bleeds were classified as minor and exhibited similar clinical characteristics in both groups. In one study of 80 patients undergoing transfemoral diagnostic cardiac catheterization with a 4F introducer sheath with hemostasis valve, where 40 patients ambulated after 2 hours and 40 after 4 hours, three patients developed bleeding in the 4-hour group, versus none in the 2-hour group^14^. With the advent of increasingly potent anticoagulant therapies and antiplatelet agents designed to reduce the incidence of periprocedural ischemic complications, a reassessment of bleeding risk is in order. Early identification of hematoma or bleeding requires knowledge, skill, and rapid intervention by the nursing team. Supervision and continued training of nurses can help ensure rapid identification and treatment of this complication, thus benefiting patient safety. 

Another complication observed only during OU stay was vasovagal reaction, which occurred in 5 (1.4%) IG participants and 4 (1.1%) CG participants. Similar results regarding this complication were observed in a study conducted at a university hospital in Australia^15^. Of the 611 patients analyzed, 35 (5.7%) developed this complication during sheath removal. Vasovagal responses triggered by anxiety or pain now occur in fewer patients, possibly due to widespread awareness of the nature of cardiac catheterization (thus reducing patient tension), better care by multidisciplinary teams, more effective sedation, and greater operator experience^1^. The malaise caused by prolonged supine positioning and immobility, compounded by difficulty urinating, pelvic discomfort, and anxiety, are predictors of vasovagal response during sheath withdrawal^15^. Within this context, shortening the duration of bed rest may reduce this complication. 

In both groups, bruising at the insertion site was the most commonly reported complication at telephone follow-up, followed by pain and hematoma. In a study^3^ of 1,446 patients undergoing diagnostic cardiac catheterization with a 6F introducer sheath with hemostasis valve, there were no major bleeding events or large hematomas, only bruising (in 10% and 21% of IG and CG patients respectively) and small hematomas (22% and 9% of IG and CG patients respectively) after discharge. Thus, the authors concluded that early ambulation was safe in their patient population^3^. 

Patients who have undergone cardiac catheterization experience restricted mobility due to arterial puncture of the catheterized limb. Back pain and discomfort secondary to immobilization are often recorded by cath lab nurses, and are the most common complaints of patients in this setting^13^. Nursing care should focus on patients' difficulties and judicious monitoring. Studies of patients undergoing interventional radiology procedures have demonstrated that duration of bed rest is associated with discomfort^2^ ^,^ ^6^ ^,^ ^12^ ^-^ ^13^ ^,^ ^16^. Indeed, discomfort and impatience have been observed in patients during the recovery period, both while in hospital and after discharge to home. Pain and discomfort was the second most common complication (complaint) at 24-hour, 48-hour, and 72-hour follow-up. A Swedish study^11^ that assessed this complication up to 3 days post-procedure found that shortening the immobilization period had beneficial effects on patient comfort and satisfaction. Prolonged rest can cause muscle weakness and fatigue due to constant pressure over the same muscle groups, and fatigue can, in turn, lead to muscle spasms and back pain. These authors also reported that reducing the duration of bed rest can reduce back pain and discomfort without increasing vascular complications. In this setting, early ambulation is a particularly relevant additional strategy to improve patient comfort after cardiac catheterization procedures. In the present sample, antiplatelet therapy and presence of comorbidities were not associated with increased risk of complications. 

In short, the strategy tested in this trial - i.e., reducing duration of bed rest after diagnostic cardiac catheterization to 3 hours - proved feasible and safe. Reduction of cath lab length of stay is consistent with a focus on resource optimization and can help meet the growing demand for procedures. Trials such as this are necessary if providers are to abandon care routines for which there is no evidence basis and change their practices, using consistent results from large-scale RCTs as a reference.

## Conclusions

Based on the results of this RCT, we conclude that reducing the duration of bed rest from 5 to 3 hours in patients undergoing transfemoral diagnostic cardiac catheterization with a 6F introducer sheath did not increase the rate of arterial puncture-related complications during OU stay or at 24-hour, 48-hour, and 72-hour telephone follow-up. Antiplatelet therapy (clopidogrel and aspirin) and presence of comorbidities (HTN, DM, and DVP) were not significantly associated with occurrence of the clinical outcomes of interest at any point during the study period. Women, regardless of group allocation, experienced more events than men at 24-hour, 48-hour, and 72-hour follow-up.
